# Loot

**Published:** 1868-01-15

**Authors:** 


### *LOOT.*

Dr. Wayne Griswold recommends (*Western Journal of Medicine*) in Scarlatina *Quinine*, *Iron*, and *Chlorate of potash*, with good nourishment, as the central measures of treatment. He prefers warm ablutions to cold, and gives for drink lemonade, ice water, or milk. Locally, to the throat:

| | |
|---|---|
| ℞.—Tinct. Ferri Chlor., - - - - | ʒ iij. |
| Potassæ Chlorat, - - - - - | ʒ j. |
| Aq. Distill., - - - - - - | ℥ iij. |

M. Apply them three times a day.

The same mixture, internally, in doses of a teaspoonful or more every four hours, alternating with moderate doses of *Quinine*. Free ventilation is insisted upon. For the Sequelæ

he advises: Free doses of *Jalap* and *Hyd. cu. cret.*, followed by *Bitart. potash*, with half a teaspoonful of the following mixture every four hours:

℞.—Fl. ext. uva ursi, - - - - ʒ vij.

Fl. ext. digitalis, - - - - ʒ j.

Sp. nit. dul., - - - - - - ℥ j.

M. Also warm ablutions.

On removal of the œdema and increase of urine., *Quinia* and *Iron* to be given in tonic doses.

A New York correspondent of the same journal writes: "At the New York Hospital and in Bellevue, the *hypodermic injection of Quinia* is used quite extensively in the treatment of Intermittent Fever and Malarial Neuralgia. At the former institution, the formula used is as follows: Take of *Sulphate of quinia*, 60 grs.; *Dilute sulphuric acid*, 40 minims; *Distilled water*, 1 fluid ounce. Mix. Make a solution, and filter with the greatest care. Thirty-five minims are equal to 4 grains of *Quinia.* Or the solution may be varied by the addition of 4 or 6 grs. of *Sulphate of morphia.* This combination renders the injection less painful." At Bellevue, an ethereal solution of *Quinia* is used, of the strength of 1 grain to 2 minims. The process of preparation is quite elaborate, and it is difficult to see where it possesses especial advantages over that of the New York Hospital, as, indeed, it is difficult to see where that possesses any real advantages over the usual methods of administration of the remedy, except in so far as it may enlist the expectant faith of the patient, which is always of great use to the physician. This latter may overcome the patient's alarm and annoyance—and subsequent liability to abscesses or erysipelas.

*Beef soup*, broths, or jellies, may be preserved from turning sour in the sick room, or elsewhere, by stirring in a few drops of the solution of *Bi-sulphite of lime.* This will not impair their taste in the least.

